# Characterization of plant growth-promoting bacteria associated with avocado trees (*Persea americana* Miller) and their potential use in the biocontrol of *Scirtothrips perseae* (avocado thrips)

**DOI:** 10.1371/journal.pone.0231215

**Published:** 2020-04-08

**Authors:** Jorge A. Tzec-Interián, Damaris Desgarennes, Gloria Carrión, Juan L. Monribot-Villanueva, José A. Guerrero-Analco, Ofelia Ferrera-Rodríguez, Dulce L. Santos-Rodríguez, Nut Liahut-Guin, Gerardo E. Caballero-Reyes, Randy Ortiz-Castro

**Affiliations:** 1 Red de Estudios Moleculares Avanzados, Instituto de Ecología, Xalapa, Veracruz, México; 2 Red de Biodiversidad y Sistemática, Instituto de Ecología, Xalapa, Veracruz, México; 3 Catedratico-CONACyT en el Instituto de Ecología A. C., Xalapa, Veracruz, México; Hainan University, CHINA

## Abstract

Plants interact with a great variety of microorganisms that inhabit the rhizosphere or the epiphytic and endophytic phyllosphere and that play critical roles in plant growth as well as the biocontrol of phytopathogens and insect pests. Avocado fruit damage caused by the thrips species *Scirtothrips perseae* leads to economic losses of 12–51% in many countries. In this study, a screening of bacteria associated with the rhizosphere or endophytic phyllosphere of avocado roots was performed to identify bacterial isolates with plant growth-promoting activity *in vitro* assays with *Arabidopsis* seedlings and to assess the biocontrol activity of the isolates against *Scirtothrips perseae*. The isolates with beneficial, pathogenic and/or neutral effects on *Arabidopsis* seedlings were identified. The plant growth-promoting bacteria were clustered in two different groups (G1 and G3B) based on their effects on root architecture and auxin responses, particularly bacteria of the *Pseudomonas* genus (MRf4-2, MRf4-4 and TRf2-7) and one *Serratia* sp. (TS3-6). Twenty strains were selected based on their plant growth promotion characteristics to evaluate their potential as thrips biocontrol agents. Analyzing the biocontrol activity of *S*. *perseae*, it was identified that *Chryseobacterium* sp. shows an entomopathogenic effect on avocado thrips survival. Through the metabolic profiling of compounds produced by bacteria with plant growth promotion activity, bioactive cyclodipeptides (CDPs) that could be responsible for the plant growth-promoting activity in *Arabidopsis* were identified in *Pseudomonas*, *Serratia* and *Stenotrophomonas*. This study unravels the diversity of bacteria from the avocado rhizosphere and highlights the potential of a unique isolate to achieve the biocontrol of *S*. *perseae*.

## Introduction

Bacteria inhabiting the roots of trees have been scarcely investigated. These microbial populations may include plant growth promoters and biological control agents [1]. In model plants, an increase in plant biomass correlated with changes in root architecture such as, increased lateral root formation and root hair development or changes in the main root growth and angle the of branching structures [2,3]. In addition, other factors, including nutrient acquisition [4,5], phytohormones production (e.g., auxins or cytokinins) or host physiological alterations may account for the probiotic properties of certain bacteria [6–9],. On the other hand, the biocontrol of pathogens and insect pests that challenge plants may be exerted by rhizobacteria through competition for nutrients, the production of antimicrobial and antifungal compounds, or the activation of plant immune responses [10–12].

The identification of rhizobacteria for their use as probiotics includes the exploration of the rhizospheres of wild and crop plants from various ecosystems. In Mexico, the growth of avocado trees in the temperate zone in Michoacán state is a growing industry for which effective and environmentally friendly management are urgently needed. Despite the importance of avocado around the world, there are just a few studies regarding its rhizosphere biology. Carzola *et al*. [13] reported that four strains of *Bacillus subtilis* were associated with avocado antifungal activity and showed apparent enzymatic and antibiotic action against phytopathogenic fungi, whereas Guevara-Avendaño *et al*. [14] identified *Bacillus* strains with the ability to control the phytopathogenic fungi *Fusarium euwallaceae* and *Graphium* sp. Méndez-Bravo *et al*. [15] described the effects of *Bacillus*, *Pseudomonas* and *Arthrobacter* isolated from the avocado rhizosphere against *Phytophthora cinnamomi* through the production of volatile organic compounds.

A major goal is to identify biological strategies to combat insect pests such as thrips, which are currently controlled by the application of highly toxic insecticides that are responsible for the decline in the populations of pollinators such as bees. Thrips diminish the fruit quality as well as avocado production rates [16]. In the United States, the avocado industry in California has estimated annual losses attributable to the avocado thrips *Scirtothrips perseae* Nakahara at $8.51 million in the short term [17]. Considering that the production of Hass avocado hybrids has grown significantly in Mexico, making it the leading producer worldwide, the severity of this pest can translate into losses of millions of dollars. In 2015, avocado production reached a total of 1,624,000 tons in Mexico [18]. Some thrips species with phytosanitary importance have been reported in Mexico, including: *Frankliniella bruneri* Watson, *Heliothrips haemorrhoidalis* Bouché, *Scirtothrips perseae* Nakahara, and *Pseudophilothrips perseae* Watson [19].

Certain bacterial genera, such as *Pseudomonas*, *Bacillus*, *Burkholderia*, *Serratia*, *Paenibacillus*, and *Streptomyces*, include species with entomopathogenic activity against different insect pests [20,21], but there is limited information regarding the biological control of avocado thrips. Therefore, searching for rhizobacteria that could be used for the biocontrol of this avocado crop pest is relevant. In this report, we isolated and identified bacteria associated with avocado trees. Since avocado is a fruit crop whose optimal yield begins five years after planting, we characterized the plant growth-promoting effects of isolated bacteria on *Arabidopsis thaliana* as well as evaluating their direct entomopathogenic activity against avocado thrips *Scirtothrips perseae*. The results obtained are relevant for the development of biotechnological applications to reduce the use of fertilizers and chemical pesticides to sustainably improve avocado growth and health.

## Material and methods

### Sampling, bacterial isolation and morphotype groups

The study/sampling collection was carried out on private land, owned by different members of APEAM (Asociación de Productores y Empacadores Exportadores de Aguacate de México), who provided us the permission to conduct the study/sampling in all locations. Soil and root samples were collected from avocado plants (*Persea americana* Miller) in the orchards of La Meza and La Meza San Angel in Taretan, El Camino 3 in Tingambato and Los Diamantes in San Juan Nuevo between October 2016 and March 2017. All these places are in Michoacán, Mexico.

The bacterial isolates were obtained from soil, rhizosphere soil, and roots (endophytic bacteria) by serial dilutions. The growth conditions were 30°C for 24–48 h on Luria-Bertani agar (LB) medium. To confirm their purity, all strains were streaked at least three times and analyzed with gram stain. The colonial macroscopic data were used to form groups by morphotype. At least 20% of each group with similar morphological characteristics and 100% of the unique strains were selected for the plant growth promotion assay with the *Arabidopsis-*bacteria coculture system.

### *In-vitro* plant growth promotion assay

For the *Arabidopsis*-bacteria coculture system, *A*. *thaliana* Col-0 seedlings, strains of *Pseudomonas aeruginosa* PAO1 (wild type strain, negative control), *P*. *aeruginosa* Δ*lasI* (*quorum-sensing* (QS) mutant strain, positive control for plant growth promotion) and selected isolated strains were used [7,22]. *Arabidopsis* seeds were surface disinfected and germinated on 0.2x Murashige and Skoog (MS) medium [23] and grown in a plant growth chamber (CARON, model 7300–50) according to conditions described by Ortiz-Castro *et al*. [7]. Seven-day-old *A*. *thaliana* seedlings (8 seedlings per plate [n = 24]) were coinoculated in direct contact with each bacterial isolate. The *A*. *thaliana* seedlings were grown for a further 7-d period by placing the plates in the growth chamber in a completely randomized design, according to the method reported by Ortiz-Castro *et al*. [22]. After the growth period, the primary root length, lateral root number, root and shoot fresh weight, and anthocyanin concentrations were measured according to the method in a previous report [24]. The lateral root density was calculated by dividing the lateral root number by the primary root length. All experiments were replicated at least twice.

### Molecular characterization and phylogenetic analysis of isolates

Genomic DNA extraction was performed by the protocol of Nicholson *et al*. [25], Cenis, [26] and Ogram *et al*. [27]. The DNA was diluted with ultrapure water (1:10). To perform molecular characterization of the isolates, the molecular markers used were the hypervariable region V3-V5 from 16S rDNA for all the bacterial isolates and the 16S rDNA gene for those isolates selected for metabolic profiling. In the PCR, the hypervariable region was amplified using the primers: V3-V5 AFRW: TACGGRAGGCAGCAG; V3-V5 BREV: CCGTCAATTCMTTTGAGTTT, while the 16S rDNA gene was amplified using the primers: 27F: AGAGTTTGATCMTGGCTCAG and R1494: CTACGGRTACCTTGTTACGAC. Both markers were amplified using the protocol described by Espinosa Asuar [28]. Each sample of extracted DNA was amplified in triplicate and purified using the GeneAll® ExpinTM PCR SV minikit according to the manufacturer's protocol. Finally, the samples were sent to Macrogen Inc. for sequencing.

The obtained sequences were compared against nucleotide databases using the NCBI BLAST algorithm at http://www.ncbi.n1m.nih.gov/blast/Blast.cgi. Triplicate sequences were edited with Applied Biosystems Sequence Scanner Software v2.0 from Thermo Fisher Scientific and processed in EGassember [29] to obtain the consensus sequence. The alignment of consensus sequences and phylogenetic analysis by maximum likelihood using bootstraping with 1,000 replicates were performed in MEGA7 [30]. 16S rDNA sequences obtained from the selected isolates for metabolic profiling were deposited in GenBank (Accession Numbers: MN098850-MN098867).

### Auxin-responsive gene expression analysis

To evaluate auxin-responsive gene expression, 5-day-old *A*. *thaliana DR5*::*uidA* [31] transgenic seedlings were coinoculated with different bacterial strains with plant growth-promoting activity and grown for an additional 6-day period. For the histochemical analysis of *β*-glucuronidase (GUS) activity, *A*. *thaliana* seedlings were incubated at 37°C in a GUS reaction buffer (0.5 mg/mL 5-bromo-4-chloro-3-indolyl-BD-glucuronide in 100 mM sodium phosphate, pH 7). The seedlings were clarified according to Malamy and Benfey [32]. At least 10 transgenic seedlings were analyzed for GUS activity by using Leica S8 APO stereoscopic microscope (Leica, Microsystem). A qualitative scale from -4 to 4 was established to compare the GUS activity of uninoculated and cocultivated *Arabidopsis* seedlings. A score of 4 represented four times more expression than the control.

### Phosphate solubilization assay

To estimate the phosphate solubilization capacity of the bacterial isolates with plant growth promotion activity, bacteria were cultured in LB medium overnight and then cultured on Pikovskaya-bromophenol blue (PKV-BMP) medium at 0.025 OD_600_ (n = 3). The PKV-BMP medium contained 10 g/L dextrose, 5 g/L Ca_3_(PO_4_)_2_, 0.5 g/L yeast extract, 0.5 g/L (NH_4_)_2_SO_4_, 0.2 g/L KCl, 0.1 g/L MgSO4 7H_2_O, 0.0001 g/L MnSO_4_, 0.0001 g/L FeSO4, and 0.025 g/L BPB. The bacterial isolates were incubated at 30° C, 180 rpm for 48 h. The cultures were harvested by centrifugation at 8,000 rpm for 5 min; the obtained supernatant was measured at 590 nm by using an Epoch2 (Biotek) spectrophotometer for the quantitative assay. The experiment was repeated twice per triplicate for each bacterial isolated evaluated.

### Entomopathogenic activity assay

We assessed the entomopathogenic activity of a selected group of bacterial strains with plant growth promotion activity with a thrips oral toxicity test. The thrips used in the test were laboratory-reared *Scirtothrips perseae* individuals reared and maintained in plastic cages with disinfected fragments of bean pods (*Phaseolus vulgaris* L.) as food and oviposition sources, at 23–25°C, relative humidity (RH) 70–75% and 12 h light. For this assay, the 20 strains were cultured in LB broth for 48 h at 28°C and adjusted by dilution until they reached 0.5 and 1.5 OD_600_. The chambers used in the assay were made with 50 mL *Falcon®* tubes cut in half and sealed with antithrips mesh (Fig 4A). The chambers were autoclaved before use. Disinfected fragments of bean pods (length <5 mm) were impregnated by immersion in bacterial solutions and were introduced into the chambers as a source of food and oviposition sites. Due to limitations on the number of laboratory-reared thrips, the 20 strains were subjected to a first screening with only four adult thrips in each chamber. In the first screening, uninoculated bean pod fragments were used as controls. The chambers were maintained at 23–25°C, 70–75% RH and 12 h light, and the number of living insects was recorded at 24, 48, 72 and 96 h. All strains were tested in triplicate. The bacterial treatments with fewer recorded live insects were selected for a second oral toxicity test. The second test was performed by placing 10 adult thrips in each chamber, using all selected strains at 1.5 OD_600_ with an insecticide mix of imidacloprid plus lamba-cyhalothrine (Turner SC^®^ Allister) at the commercial dose as a positive control and uninoculated bean pod fragments as negative control. The selected strains were tested in triplicate, the chambers were maintained under the previously described conditions and the number of live insects was recorded at 24, 48, 72, 96 and 120 h.

### Chemical profiling of plant growth-promoting activity strains

A selected group of bacterial strains with plant growth promotion activity in *A*. *thaliana* were cultured in 300 mL of LB broth with 0.5 OD_600_ and grown for 48 h at 30°C and 150 rpm. The cultures were centrifuged at 12,000 rpm and the supernatant was lyophilized (Labconco FreeZone^™^ Freeze-Dry Systems 4.5L). Extractions were performed by sonication for 3 periods of 5 min each using methanol (1:10 v/v) in an ultrasonic bath (CPXH series) at 20°C. The extracts were centrifuged at 6500 rpm for 5 min at 20°C. The supernatant was collected, and the methanol was removed under vacuum pressure through a rotaevaporator (Büchi® Rotavapor® RII). The samples were analyzed in a Waters ultrahigh-performance liquid chromatographic (UHPLC) class I system coupled to a Synapt HDMi mass spectrometer (see Supplementary information). The data were acquired and processed with MassLynx (Waters^TM^, version 4.1) and MarkerLynx (Waters^TM^, version 4.1) software to determine the distinct chemical biomarkers through orthogonal partial least squares discriminant analysis (OPLS-DA).

### Data analysis

Data were analyzed with the R programming language [33]. For the plant growth promotion assay, a cluster analysis of the primary root length, lateral root number, root and shoot fresh weight, and anthocyanin concentration was performed with all the assessed bacterial isolates. The data were normalized (*Scale*) and grouped with the *K-means* method in three blocks. For the dendrogram construction, a distance matrix was calculated using the "*Euclidian*" method based on the grouped data and graphed with the "*complete*" method of the *cluster* package [34]. Group formation was evaluated statistically through similarity analysis (ANOSIM) using the *vegan* package [35]. Chemical profiling data were visualized with a heatmap graph, and for the proposed candidate compounds, the METLIN metabolomics database (https://metlin.scripps.edu) was consulted. The mass/charge (m/z) ratio of the quasi-molecular ions and the fragmentation pattern were compared with the Metlin database, and the maximum error allowed was five (Δppm). The results of the entomopathogenic bioassay with the thrips were analyzed through survival analysis with the log-rank test from the *survival* package [36,37]. The multiple paired comparison of the curves was performed with the *survminer* package [38]. Graphs were constructed using the *ggplot* [39] and *ggpubr* [40] packages.

## Results

### Bacteria associated with avocado trees show plant growth-promoting activity in Arabidopsis thaliana

**Fig 1:**
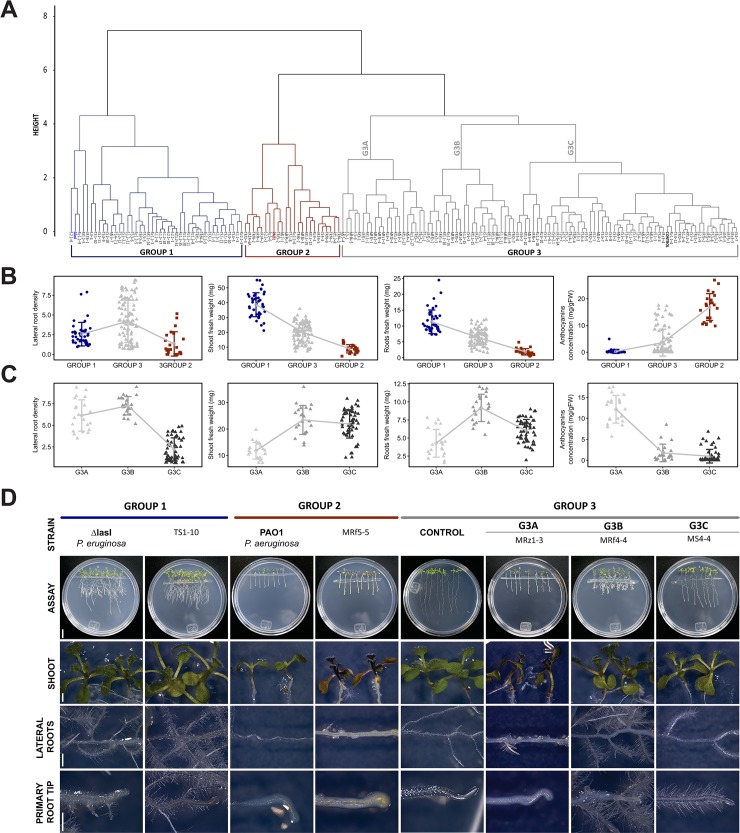
Avocado rhizosphere bacteria with plant growth-promoting activity in *Arabidopsis thaliana*. Cluster analysis of bacterial isolates associated with *Persea americana* Miller evaluated in the *Arabidopsis*-bacteria coculture system, ANOSIM, p<0.05, R = 0.67. **(A)** Dendrogram resulting from the cluster analysis of the 162 selected isolates. **(B)** Analysis of the grouping pattern of Groups 1, 2 and 3 considering the arithmetic means per group for each variable. **(C)** Grouping patterns of subgroups G3A, G3B, and G3C. The points represent each of the observations, and the bars represent the standard deviation. **(D)** Representative images of the growth of *Arabidopsis* seedlings inoculated with strains associated with the different groups and subgroups. Control plants are shown as uninoculated or inoculated with *P*. *aeruginosa* PAO1 (wild-type strain, pathogenic) and *P*. *aeruginosa* Δ*lasI* (QS mutant strain, promoter). Scale bar: Petri dishes = 1 cm; plant zones = 3 mm.

A total of 446 bacterial isolates were obtained from the different soil, rhizosphere and root samples. We evaluated the *in vitro* effect of the 162 selected strains by using an *Arabidopsis-*bacteria coculture system to screen the bacterial growth promotion activity on plants. The dendrogram in Fig 1A was obtained from cluster analysis and differentiated the treatments into three groups: **Group 1 (G1)** contained 42 isolates and the positive control *P*. *aeruginosa* Δ*lasI*, **Group 2 (G2)** contained 23 isolates and the negative control *P*. *aeruginosa* PAO1 and **Group 3 (G3)** contained 97 isolates and the uninoculated control. Statistically significant differences were found among groups through an analysis of similarity (ANOSIM, p<0.05, R = 0.67).

When plotting the group means of the variables measured in the three groups (Fig 1B), some patterns were observed. The isolates associated with *P*. *aeruginosa* Δ*lasI* (**G1**) induced the highest shoot and root fresh weight values and a lower anthocyanin concentration in the inoculated seedlings while the isolates grouped with *P*. *aeruginosa* PAO1 in **G2** showed the opposite pattern (Fig 1B**)**. Plant growth promotion by **G1** isolates is represented by TS1-10 (Fig 1D), whereas isolates from **G2** with a deleterious effect are shown by MRf5-5 (Fig 1D).

**G3** isolates were associated with control as "neutral" strains. As shown in Fig 1C, we found three types of bacteria in **G3**. The first type, isolates grouped in **G3A**, caused some type of damage in the plant (necrosis, highest mean for anthocyanin concentration), but induced many lateral roots and an inhibition in the growth of the primary root tip. These effects are shown by the *Arabidopsis* seedlings coinoculated with the MRz1-3 strains (Fig 1D). The second group, **G3B** strains, in addition to producing a short primary root length, stimulated lateral root formation and enhanced shoot fresh weight without anthocyanin induction, for example, MRf4-4 (Fig 1D). The third group, **G3C**, were the isolates most similar to the uninoculated control. Representative photographs of the growth of *Arabidopsis* coinoculated with the MS4-4 strains from this subgroup were compared with photographs of the other subgroups (Fig 1D).

The following experiments were focused on the strains in the G1 and G3B groups because of their clearly observed plant growth-promoting effects.

### Molecular characterization of bacteria with plant growth promoting activity

**Fig 2:**
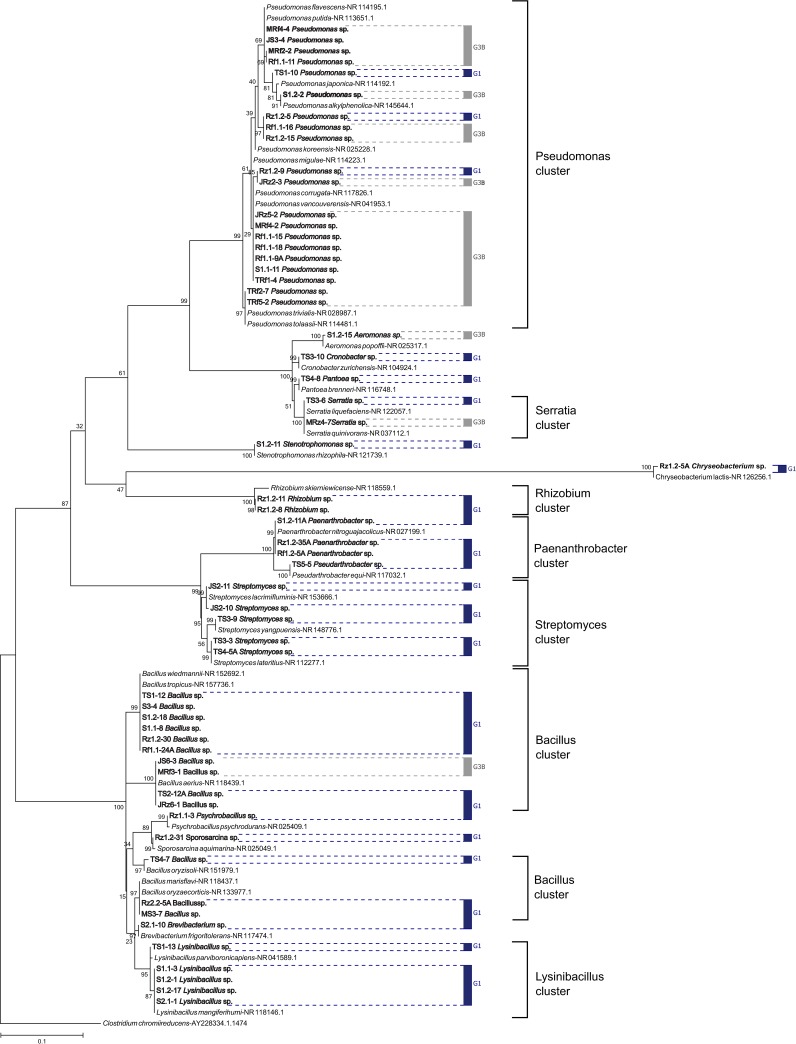
Phylogenetic relationships among G1 and G3B strains, including NCHB references, were inferred using the maximum likelihood method. The percentage of the replicate trees in which the associated taxa clustered together in the bootstrap test (1,000 runs) is shown next to the branches. The tree is drawn to scale. G1 is indicated with blue lines, and G3B is indicated with gray lines.

The bacterial isolates were molecularly identified by analyzing the hypervariable region V3-V4 from 16S rDNA. Sixteen genera were identified from the **G1** and **G3B** groups. The **G1** *Bacillus* group comprised 10 strains, while **G3B** mostly consisted of *Pseudomonas*. In general, a greater richness of bacterial genera was found in the **G1** group than in the **G3B** group. The phylogenetic tree presented in Fig 2 shows 88% **G1** and 100% **G3B** strains. Notably, although the dominant genera were different for each group, there was not a strict taxonomic separation between the two groups analyzed. The genera *Bacillus*, *Pseudomonas* and *Serratia* were found in both groups.

Although this study focused on bacterial isolates with plant growth-promoting activity (PGPB), at least 15% of the isolates from the other groups were sequenced. The phylogenetic relationships among the **G2**, **G3A** and **G3C** groups are shown in S1 Fig.

### Plant growth-promoting activity is regulated through phosphate solubilization and the modulation of auxin-related gene expression in Arabidopsis

One indirect mechanism of action of PGPB is inorganic phosphate solubilization, allowing P uptake in the plant. Therefore, we analyzed the capability of strains with plant growth promotion activity to solubilize tricalcium phosphate by using Pikovskaya-bromophenol blue (PKV-BMP) medium. Interestingly, in comparison with the control, five strains from *Pseudomonas* sp. (Rf1.1-9A, TRf5-2, TS1.10, TRf2-7, JRz5-2) and one strain from *Brevibacterium* sp. (S2.1–10) showed the highest phosphate solubilization activity, while strains *Lysinibacillus* sp. S1.2–17, *Pseudomonas* sp. Rz1.2–5, and *Streptomyces* sp. JS2-10 showed low phosphate solubilization activity (S2 Fig).

**Fig 3:**
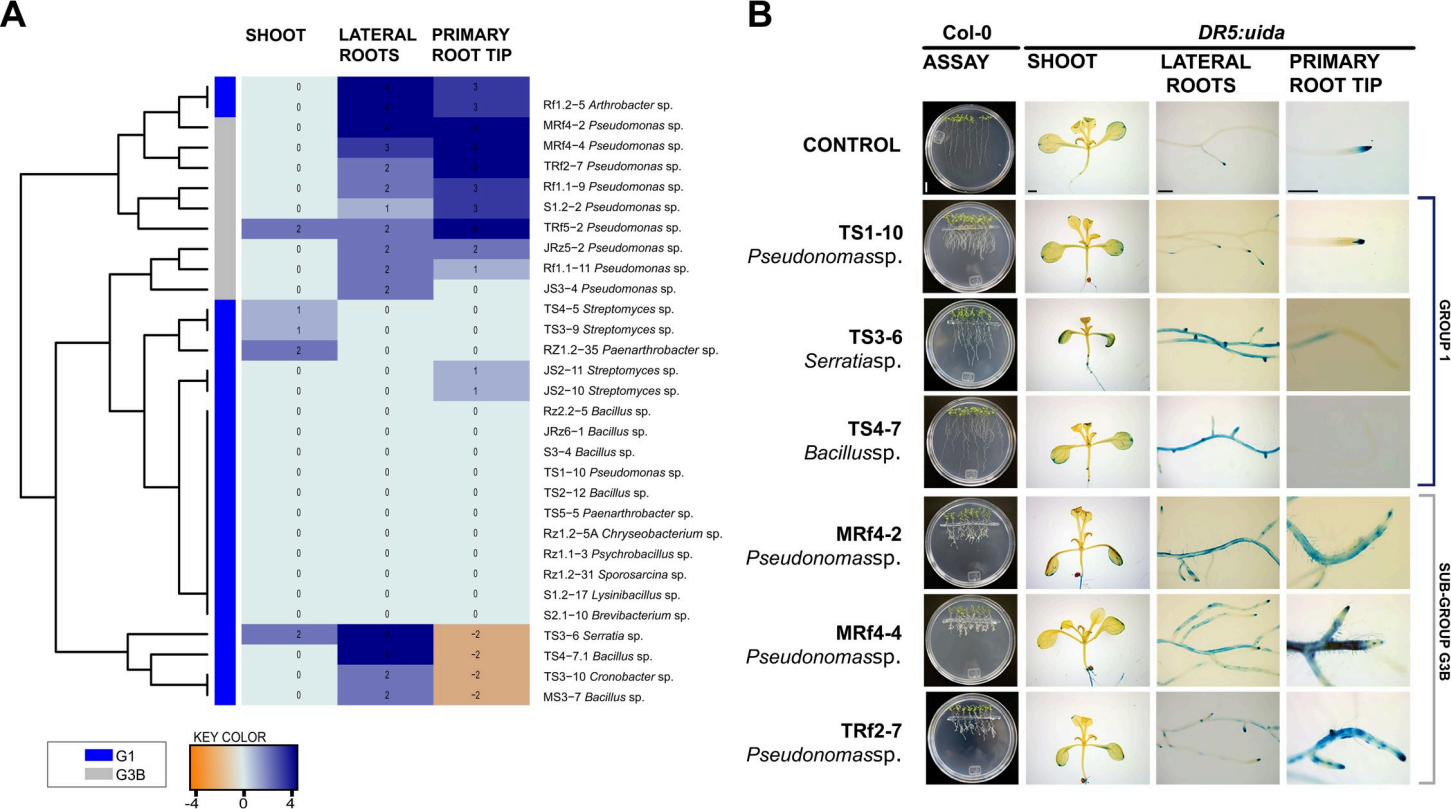
Effect of some bacterial isolates from G1 and G3B on the *DR5*::*uidA* reporter gene for auxin responses in *Arabidopsis thaliana*. **(A)** Qualitative analysis of *uidA* gene expression in *A*. *thaliana* transgenic lines inoculated with bacterial isolates. Values were obtained by comparing the treatments with the control without inoculation. A scale with a range of 4 to -4 was established for this analysis. Higher values and a more intense blue color indicate higher expression compared to that in the control. The orange color indicates an absence of gene expression (NE). **(B)** Representative photographs of the shoot, lateral root zone and meristem of the primary root of transgenic *DR5*::*uidA* seedlings inoculated with bacterial strains. Scale bar: Petri dishes = 1 cm, plant zones = 500 μm.

Several effects shown by the PGPB from the G1 and G3B groups are related to auxin-like activity. *Arabidopsis* transgenic lines of *DR5*::*uidA* were inoculated with 21 selected strains, and *uidA* gene expression was analyzed (Fig 3). The heat map constructed showed a clear differentiation between strains **G1** and **G3B** (Fig 3A) on the *DR5*::*uidA* gene marker. Ten strains (*Serratia* sp. TS3-6; *Bacillus* sp. TS4-7.1; *Pseudomonas* sp. MRF4-2, MRF4-4, Rz1.2–5, Rf1.1–9, S1.2–2, TRf5-2 and TRF2-7; *Arthrobacter* sp. Rf1.2–5) induced the highest production of auxin in lateral root zone and/or in the primary root meristem (Fig 3A and 3B). Interestingly, *Serratia* sp. TS3-6, *Bacillus* sp. TS4-7 and *Cronobacter* sp. TS3-10 had an unusual lack of auxinic gene expression in the primary root tip, in contrast with the high expression in the lateral root zone.

### Plant growth-promoting bacteria associated with avocado show entomopathogenic activity

**Fig 4:**
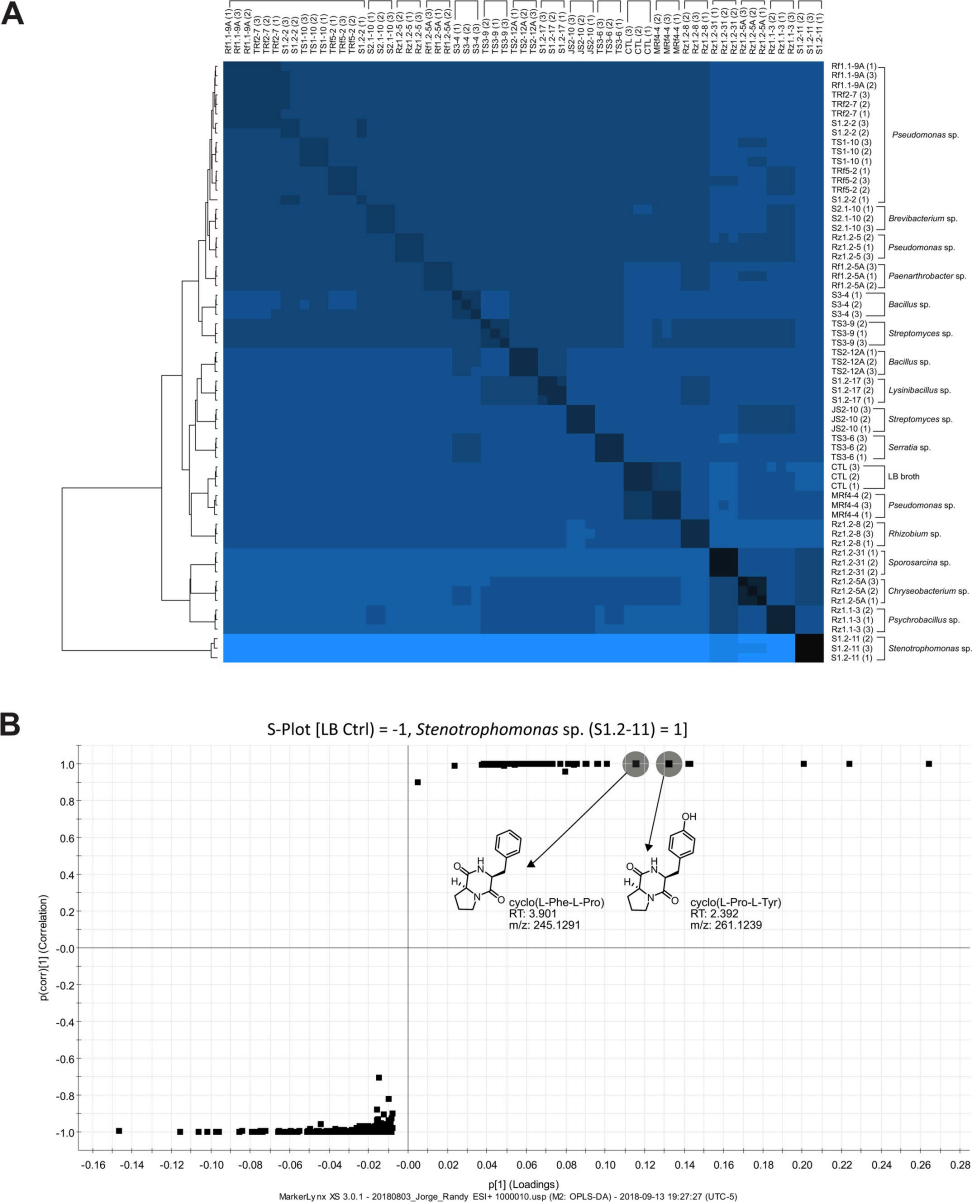
Entomopathogenic activity of plant growth-promoting rhizobacteria from avocado. (**A**) Representative diagram of the chamber design of the *in-vitro* entomopathogenic activity assay per strain. The image shows the number of repetitions per concentration of bacterial strain analyzed, as well as the number of insects used per chamber. (**B**) Oral toxicity assay of seven of the twenty strains evaluated with *S*. *perseae*. Thrips were exposed to the strains for 96 h in a bioassay chamber. Bean pod slices impregnated with two bacterial concentrations were used as food. Data for the commercial control and the control with no inoculation are presented in red and black, respectively. The median is shown as a bar inside the boxes, and the whiskers indicate to the range of the data.

Twenty bacterial strains with the best plant growth promotion characteristics were evaluated in an entomopathogenic bioassay with laboratory-reared avocado thrips *Scirtothrips perseae*. As a first approach, the 20 strains were used in bacterial solutions at 0.5 and 1.5 OD_600_ to test their oral toxicity on *S*. *perseae* for four days (96 h, Fig 4A). To compare the number of dead insects in the different treatments, a survival analysis with a *log-rank* test was performed. Statistically significant differences were found both when comparing the curves with respect to the OD concentrations [*X*^*2*^ = 83.5, p<0.05] and when only the strains were considered [*X*^*2*^ = 70.9, p<0.05]. In the multiple paired comparisons of the curves with *p* values adjusted with the BH method, only *Chryseobacterium* sp. Rz1.2-5A presented a difference at the margin of statistical significance (p = 0.058) compared with the control. Although no significant differences were recorded between the strains and the control, some tendencies were observed. After 48 h, no live insects were counted in six of the 20 strains analyzed (S3 Fig). In the chambers where the insects were exposed to *Chryseobacterium* sp. Rz1.2-5A, regardless to the concentration, the insects died at 24 h, while in the control, live insects were recorded until 72 h (S3 Fig). From this first screening, the three bacterial strains with the smallest numbers of live thrips recorded were selected for a second oral toxicity test with 120 h of evaluation. In the second test, a higher number of thrips and an insecticide mix of imidacloprid plus lamda-cyhalotrine (chemical control) were used. The selected strains were *Chryseobacterium* sp. Rz1.2-5A, *Pseudomonas* sp. Rf1.1-9A and *Pseudomonas* sp. TRf5-2, in which <1 live thrips were recorded after 24 h at OD_600_ 1.5 (S3 Fig). In the second test, dead thrips were recorded at 24 h in *Pseudomonas* sp. Rf1.1-9A treatment, even earlier than in the chemical control, and the survival probability was reduced to <80%. Interestingly, at 72 h in *Chryseobacterium* sp. Rz1.2-5A treatment the thrips survival probability was reduced to 50%, the same level observed in the chemical control (Fig 4B). To compare the number of dead thrips in the different treatments, a survival analysis with a log-rank test comparison was performed, and significant differences were detected between the control and all treatments, including the chemical control (*X*^*2*^ = 65.9, df = 4, p<0.0001; Fig 4B). After a multiple pairwise comparisons of the curves, *Chryseobacterium* sp. Rz1.2-5A showed a similar survival curve to the chemical control (p = 0.655), suggesting the same effectiveness level.

### Chemical profiling of plant growth-promoting bacteria

**Fig 5:**
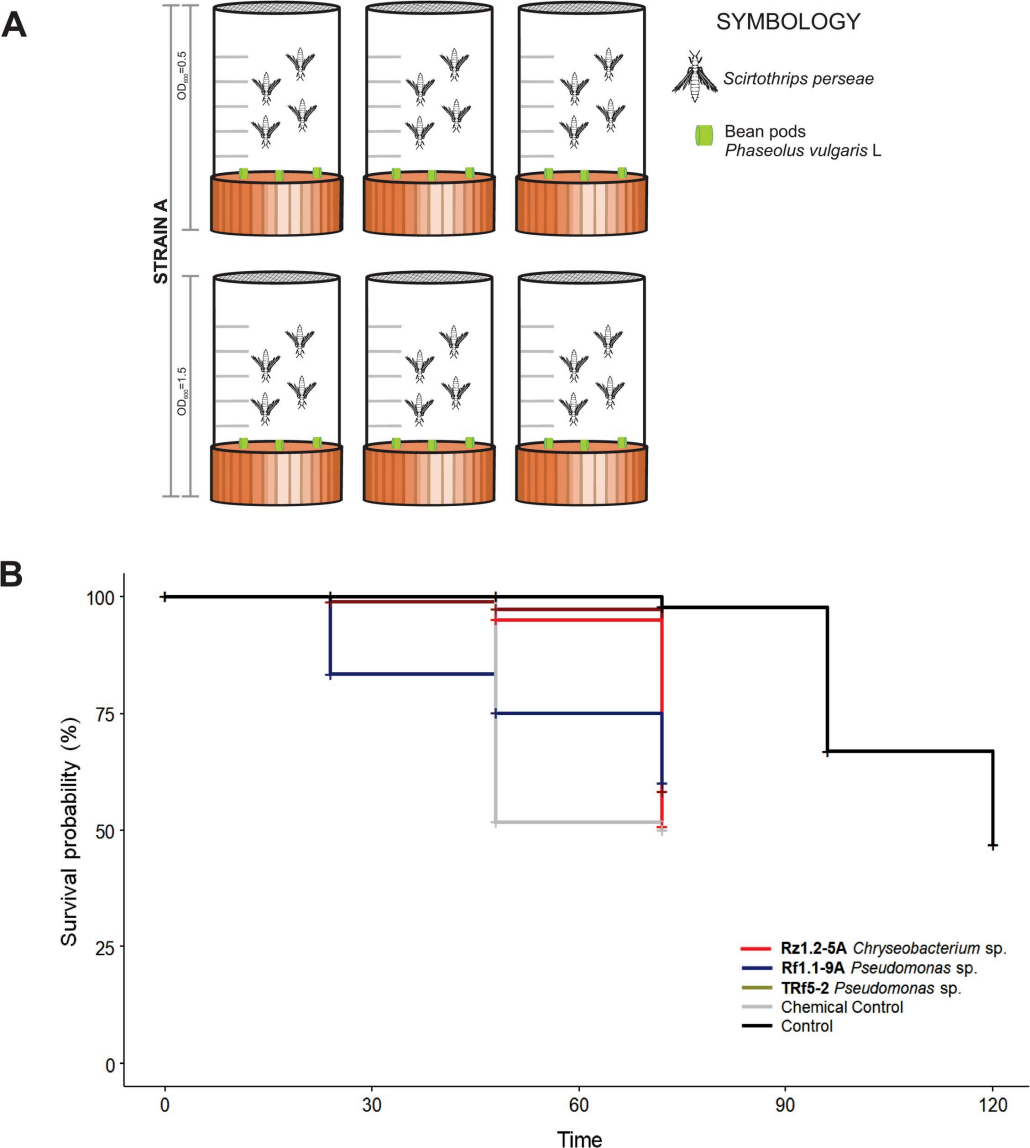
Chemical profile of avocado rhizosphere bacteria with plant growth-promoting activity. **(A)** Heat map representation of the chemical profile of the twenty plant growth promoting rhizobacteria strain extracts. Cluster relationships are presented through the dendrogram on the left side of the heat map. This graph was constructed considering analytical triplicates. LB broth was used as the culture medium and is indicated in the graph as CTL. **(B)** S-plot analysis of the differential compounds produced between *Pseudomonas* MRf4-4 and a set of 6 other *Pseudomonas* strains.

Considering the chemical natures of the compounds produced by different bacteria, chemical profiling of 20 bacterial strains was performed to determine whether their plant growth-promoting characteristics could be reflected at the metabolic level. The 20 bacterial strains subjected to chemical profiling were chosen based on their plant growth promotion characteristics and their potential as thrips biocontrol agents. The heat map in Fig 5A shows the chemical profile for each of the selected strains, and it is possible to differentiate some bands or groups. Interestingly, the bands at the ends (top and bottom) of the heat map showed differences compared with the control. The strains inserted in the middle band seem to be similar to the LB broth profile (CTL). At the bottom, the *Stenotrophomonas* sp. S1.2–11 band presents a completely different chemical profile compared to all others, while at the other end a *Pseudomonas* sp. (Rf1.1-9A, Rz1.2–5, TRf2-7, S1.2–2, TS1-10, TRf5-2) cluster is located. Of the seven strains of *Pseudomonas* included in the analysis, only *Pseudomonas* sp. MRf4-4 is separate from this group (Fig 5A).

Exploring the chromatograms and spectrometric fingerprints allowed the tentative identification of a group of cyclodipeptides (CDPs, **Table 1**), compounds with auxin-like activity [7]. From the *Pseudomonas* cluster we identified cyclo (L-His-L-Leu). This same CDP was found for *Serratia* sp. TS3-6 as well (**Table 1**). Regarding *Stenotrophomonas* sp. S1.2–11, differential analysis with orthogonal partial least squares discriminant analysis (OPLS-DA) was performed to compare this unique strain to the LB broth (control). The S-plot of this analysis is presented in Fig 5B, where points located at negative values represent the differential compounds or fragments of the LB broth, while points at positive values indicate the differential compounds or fragments of the *Stenotrophomonas* sp. strain. This analysis identified two CDPs: cyclo(L-Pro-L-Tyr) and cyclo(L-Phe-L-Pro). The fragment pattern matches the databases for these three CDPs (mass spectra in S4 Fig). These results suggest the possible role of cyclodipeptides in plant growth promotion and entomopathogenic activity.

**Table 1:** Identification of cyclodipeptides from bacteria with plant growth promoting activity.

STRAIN	ID	GROUP	m/z	RT (min)	QMI	ERROR (ppm)	CDP
***Pseudomonas sp*.**	S1.2–2	G3B	251.15	1.343	[M+H]^+^	4.4	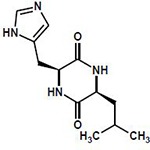
	Rf1.1-9A	G3B	251.15	1.343	[M+H]^+^	2.8	
	TRf2-7	G3B	251.15	1.326	[M+H]^+^	1.2	
	TRf5-2	G3B	251.15	1.343	[M+H]^+^	1.2	
	Rz1.2–5	G1	251.15	1.363	[M+H]^+^	1.9	**cyclo(L-His-L-Leu)**
	TS1-10	G1	251.15	1.363	[M+H]^+^	2.3	C_12_H_18_N_4_O_2_
***Serratia sp*.**	TS3-6	G1	251.15	1.380	[M+H]^+^	2.8	M: 250.14297
***Stenotrophomonas sp*.**	S1.2–11	G1	261.12	2.392	[M+H]^+^	1.9	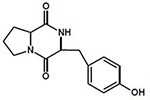
							**cyclo(L-Pro-L-Tyr)**
							C_14_H_16_N_2_O_3_
							M: 260.116089
			245.12	3.901	[M+H]^+^	0.8	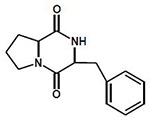
							**cyclo(L-Phe-L-Pro)**
							C_14_H_16_N_2_O_2_
							M: 244.121185

ID: code name, m/z: mass-charge ratio, RT: retention time, QMI: quasi-molecular ion, CDP: cyclodipeptide.

## Discussion

Several studies have used the *Arabidopsis thaliana* plant model to evaluate the beneficial, pathogenic or neutral effects of microorganisms associated with plants and as a rapid system for identifying or characterizing plant growth-promoting bacteria [6–8,15,22,41,44,63]. Avocados are large forest trees, often over 20 m tall, that exhibit rhythmic growth with two or more flushes of shoot growth per year, alternating with periods of rest, and that reach optimal yields until five years after planting [42], while *A*. *thaliana* completes its life cycle in 8–12 weeks from germination to harvesting. In this study, we used the *A*. *thaliana* model to evaluate the plant growth-promoting effects of bacteria isolated from avocado trees in a shorter time. The notable high biomass of *A*. *thaliana* seedlings after inoculation with strains from Group 1 is a characteristic commonly attributed to several plant growth-promoting rhizobacteria (PGPR) of *Bacillus and Pseudomonas* strains in *Phaseolus vulgaris* [41], *Solanum lycopersicum* L., *Amaranthus* sp. [43] and *Arabidopsis* [44]. This suggest that **G1** could be considered plant growth-promoting bacteria.

In Group 2, the high anthocyanin concentration it was a defining characteristic. The role of anthocyanins as indicators of stress in plant-microbe interactions is well understood. Specifically, transcriptomic studies have shown an increased expression of genes related to anthocyanin biosynthesis during pathogen attack [45,46]. Himemo *et al*. [47] showed the correlation of this type of compound with a defense mechanism to prevent cell death in the shoots of infected plants. The effects observed in plants inoculated with strains associated with negative control *P*. *aeruginosa* PAO1 in **G2** reflect phytopathogenic or opportunistic bacteria causing some kind of damage or stress in the plant and compromising foliar and radicular development, since the plants with the lowest lateral root number and shoot weight were recorded in this group.

In this sense, the high anthocyanin concentrations recorded with **G3A** strains denote a pathogenic effect in *Arabidopsis* seedlings; therefore, the high lateral root number values for these strains imply an opposite effect. Dovana *et al*. [48] described how some endophytic pathogenic fungi might reduce cell elongation by producing more cells per unit volume when the increase in dryness is not related to fresh weight and noted the involvement of IAA in this effect. This could explain how **G3A** strains showed high values for lateral root density and low shoot and root fresh weights. Thus, we propose that the **G3A** group contains phytopathogenic strains. In the case of the strains grouped in **G3B**, the effects observed in *Arabidopsis* plants are similar to those in **G1**, except for a primary root shortening, which can be explained by the concentration of the inoculum. Shi *et al*. [49] and Kwon *et al*. [50], who worked with *Serratia marcescens* and *Paenibacillus polymyxa*, respectively, indicated that low concentrations of inoculum cause a growth promotion effect, while a high concentration inhibits the elongation of the primary root. Therefore, the strains of the **G3B** group can be considered growth promoters, and a larger primary root could be achieved by regulating the inoculum concentration. Finally, the **G3C** group strains associated with the uninoculated control can be considered neutral strains that have a null or reduced effect on the inoculated plants within the framework of the assay performed.

We molecularly identified the strains with plant growth-promoting characteristics from the **G1** and **G3B** groups. The identified taxonomic positions have been reported in other studies describing the microbial community of avocado tree soil [51] and the rhizospheres of other Lauraceae species [52]. Recently, Méndez-Bravo *et al*. [15] described seven bacterial isolates of the genera *Bacillus*, *Pseudomonas* and *Arthrobacter* (the old name of several *Paenarthrobacter* species) with PGPR activity through the production of volatile organic compounds in *Arabidopsis* seedlings. All these genera were identified in our study. *Bacillus* and *Pseudomonas* were the two main genera we found, with *Pseudomonas* being better represented in group **G3B** and *Bacillus* in group **G1**. In general, all the genera identified include at least one species that has been reported in other studies as s plant growth promoter [53–60].

The presence of *Pseudomonas* in all groups represents an interesting point not only for the versatility of its effects in many plant systems, but also because it can be identified as a good auxin gene expression promoter and phosphate solubilizer when it is related to plant growth promotion activity. Most of the *Pseudomonas* strains that induced high *DR5*::*uidA* gene expression for auxin biosynthesis belonged to the subgroup **G3B**, so the expression of this particular gene could be directly associated with short primary root length, the main characteristic that distinguishes this group from **G1**. Strain concentration has been noted to directly affect the primary root length as in the **G3B** strains. Some authors attribute this effect to indole-3-acetic acid (IAA), a phytohormone that acts as a repressor of the elongation of the primary root at high concentrations [61,62]. We found this relationship between reduced primary root elongation and high auxin expression in **G3B** group.

The modulation of auxin expression by plant growth promoter strains from G1 is more heterogeneous than that of strains of **G3B**, which seems to be related to the higher diversity of the bacterial strains in G1. Moreover, some authors have described nutritional pathways such as phosphate solubilization, nitrogen fixation and siderophore production [63] as well as auxin-independent mechanisms [41]. In the same way, 2,3-butanediol, acetoin and other volatile organic compounds from rhizobacteria are reported to enhance plant growth through auxin-independent hormonal pathways [64].

Considering the plant growth-promoting characteristics described for the strains in the **G1** and **G3B** groups, we studied the potential of a select group of strains to act as biocontrol agents through an entomopathogenic bioassay against *S*. *perseae*, one of the most harmful avocado pests. Notably, *Chryseobacterium* sp. Rz1.2-5A had an entomopathogenic effect on the thrips, showing a similar efficiency level as the chemical control. In a detailed search of the literature, we did not find publications about bacteria in the *Chryseobacterium* genus showing entomopathogenic activity. The closest report was one published by Yi *et al*. [65], who described a strain of the genus *Flavobacterium*, which is phylogenetically close to *Chryseobacterium*, showing entomopathogenic activity against the larvae of *Spodoptera exigua*, or commonly known as the settled worm, which is a pest of multiple crops.

Interestingly, in exploring the chemical profiles presented in the heatmap we identified the same presumed CDPs for almost all strains of *Pseudomonas*, except MRf4-4, the only strain in the heatmap not grouped with its congeners. The similarity found in the heatmap between the MRf4-4 and LB medium profiles (control), indicates the need to explore longer incubation times or larger extraction volumes to corroborate the capacity of this strain to produce the CDPs identified. Two others presumed CDPs were identified in *Stenotrophomonas* sp. and located as differential metabolites compared to those in the control. In a recent study, Song et al. [66] evaluated plant immune activation through seed defense biopriming (SDB) by using metabolites from root-associated *Bacillus* spp. isolates and found that *B*. *gaemokensis* strain PB69 increased the mortality of insect the pest *Spodoptera litura* fed with cucumber or pepper tissues previously treated with *B*. *gaemokensis*. They identified cyclo(L-Leu-L-Pro) as the compound responsible for SDB activity. Previously, Ortiz-Castro et al., [7] described three CDPs, cyclo(L-Pro-L-Tyr), cyclo(L-Pro-L-Phe) and cyclo(L-Pro-L-Val), from *Pseudomonas aeruginosa* that acted as auxin-like compounds responsible for plant growth promotion in *Arabidopsis* seedlings. This is relevant not only for understanding the possible participation of these compounds in plant growth promotion and pest biocontrol but could also be related to other important roles of the bacterial strains during plant-microbe interactions. The current study provides a basis for the potential application of rhizobacterial strains in avocado.

## Supporting information

S1 Fig Phylogenetic relationships of the G2, G3A and G3C strains, including NCHB references, were inferred using the maximum-likelihood method. The percentage of the replicate trees in which the associated taxa clustered together in the bootstrap test (1,000 runs) is shown next to the branches. The tree is drawn to scale. G2 is indicated with red lines, while G3A and G3C are indicated with gray lines.
(EPS)

S2 Fig Effects of bacterial isolates with plant growth promotion activity on phosphate solubilization. Bacterial isolates were cultured on Pikosvskaya-bromophenol (PKV-BMP) medium with a 0.025 OD_600_ at 30°C, 180 rpm for 48 h. The data represent the means ± SD (n = 3). The experiment was replicated two times with similar results.
(TIFF)

S3 Fig Oral toxicity assays of twenty strains evaluated in *S*. *persea*. Thrips were exposed to the strains for 96 h in a bioassay chamber. Bean pod slices impregnated with two bacterial concentrations were used as food. Data for the commercial control and the control with no inoculation are presented in red and black, respectively. The median is shown as a bar inside the boxes and the whiskers indicate to the range of the data.
(EPS)

S4 Fig Mass spectra of the compounds obtained from methanolic extracts. **(A)** Compound with RT = 3.944 and m/z = 245.1295 obtained from the methanolic extract of *Stenotrophomonas* sp. S1.2–11. The fragmentation pattern corresponds to cyclo(L-Phe-L-Pro). **(B)** Compound with RT = 2.40 and m/z = 261.1243 obtained from the methanolic extract of *Stenotrophomonas* sp. S1.2–11. The fragmentation pattern corresponds to cyclo(L-Pro-L-Tyr). **(C)** Compound with RT = 1.343 and m/z = 251.1515 obtained from the methanolic extract of *Pseudomonas* sp. Rf1.1-9A. The fragmentation pattern corresponds to cyclo(L-His-L-Leu).
(EPS)

S1 Data Conditions of chemical profile analysis. (DOCX)
