# Supplementary material for: Characterization of plant growth-promoting bacteria associated with avocado trees (Persea americana Miller) and their potential use in the biocontrol of Scirtothrips perseae (avocado thrips)
Source: PLoS One. 2020 Apr 8;15(4):e0231215. doi: 10.1371/journal.pone.0231215 (PMC7141680; doi:10.1371/journal.pone.0231215)
Supplement: S1 Data — (DOCX) [file pone.0231215.s005.docx]

**Supplementary methods**

**Conditions of chemical profile analysis**

The chromatography was carried out on an Acquity BEH column (1.7 μm, 2.1 x 50 mm) with column and sample temperatures of 40 °C and 15 °C, respectively. The mobile phase consisted of (A) water and (B) acetonitrile, both with 0.1% formic acid (SIGMA). The gradient conditions of the mobile phases were 0-13 min linear gradient 1 -80% of B, 13-14 min isocratic at 80% of B, and 14-15 min linear gradient 80-1% of B (total run time 20 min). The flow rate was 0.3 mL/min, and 1 µL of extract was injected. The mass spectrometric analysis was performed with an electrospray ionization source in negative and positive mode with capillary, sampling cone and source offset voltages of 3,000, 40 and 80 V, respectively. The source temperature was 100 °C, and the desolvation temperature was 20 °C. The desolvation gas flow was 600 L/h, and the nebulizer pressure was 6.5 bar. Leucine-enkephalin was used as the lock mass (556.2771, [M+H]^+^; 554.2615, [M-H]^-^). The conditions used for MS analysis were as follows: mass range 50-1200 Da, function 1 CE, 6 V, function 2 CER 10-30 V, scan time 0.5 sec. The retention times and the protonated masses were generated at a noise threshold of 10, 000 counts, and smoothing was applied.
